# Medical Students' Longitudinal Learning With Older Adults: Geriatrics Education Mentor Program

**DOI:** 10.1093/geroni/igaa057.046

**Published:** 2020-12-16

**Authors:** Marilyn Gugliucci, Victoria Thieme

**Affiliations:** University of New England College of Osteopathic Medicine, Biddeford, Maine, United States

## Abstract

The University of New England College of Osteopathic Medicine (UNECOM) Geriatrics Education Mentor (GEM) Program is in its sixth year. It's a joint project between older community living adults and 2nd year medical students within the Osteopathic Clinical Skills course. The GEM program goal is to foster understanding and importance of the person/patient-provider relationship. Pairs of osteopathic medical students (N=87 +/- 5) were assigned to an older adult GEM (mentor) volunteer (N=87 +/-5) living within a 50 mile radius of UNECOM. Snowball sampling was used to recruit the GEMs; student participation is required. Four (4) home visits were conducted over 9 months with an assigned GEM; each visit included a new assignment. Students observed, summarized, and recorded experiences communicated by the GEM for each cumulative assignment. Data from assignments were “graded” and content analyses of open ended evaluation/summary questions were conducted. This program is UNE IRB approved. Student pairs completed all assignments. Assignments analysis on Blackboard revealed that students developed respect and awareness of life and medical experiences of the GEM; expressed understanding of the GEM’s life experiences and goals for what “matters most”. Relationships with older adults were established while students maintained professionalism and succeeded in competent and confident interactions. Documentation review from the 4 assignments aided in building students’ geriatrics knowledge, attitudes and skills. Pre-clinical medical education tends to be episodic; providing an opportunity to establish longitudinal assignments over the course of the academic year with the same older adult augments relationship skills and learning in geriatrics.

